# Effects of COVID-19 on Arrhythmia

**DOI:** 10.3390/jcdd9090292

**Published:** 2022-09-02

**Authors:** Yujia Zhan, Honghua Yue, Weitao Liang, Zhong Wu

**Affiliations:** Department of Cardiovascular Surgery, West China Hospital, Sichuan University, Chengdu 610017, China

**Keywords:** COVID-19, SARS-CoV-2, arrhythmia, cardiovascular disease, coronavirus

## Abstract

The World Health Organization announced that COVID-19, with SARS-CoV-2 as its pathogen, had become a pandemic on 11 March 2020. Today, the global epidemic situation is still serious. With the development of research, cardiovascular injury in patients with COVID-19, such as arrhythmia, myocardial injury, and heart failure, is the second major symptom in addition to respiratory symptoms, and cardiovascular injury is related to the prognosis and mortality of patients. The incidence of arrhythmia in COVID-19 patients ranges from 10% to 20%. The potential mechanisms include viral infection-induced angiotensin-converting enzyme 2 expression change, myocarditis, cytokine storm, cardiac injury, electrophysiological effects, hypoxemia, myocardial strain, electrolyte abnormalities, intravascular volume imbalance, drug toxicities and interactions, and stress response caused by virus infection. COVID-19 complicated with arrhythmia needs to be accounted for and integrated in management. This article reviews the incidence, potential mechanisms, and related management measures of arrhythmia in COVID-19 patients.

## 1. Introduction

On 31 December 2019, severe acute respiratory syndrome caused by a novel coronavirus was officially reported for the first time in Wuhan, Hubei Province, China [1]. The new disease spread rapidly at an astonishing rate. As of 5 March 2022, there were nearly 600 million cumulative cases and nearly 6.5 million deaths worldwide, and these data are still increasing [2]. The pathogen of the disease was identified by the World Health Organization and named severe acute respiratory syndrome coronavirus 2 (SARS-CoV-2).

The most common symptoms of people infected with SARS-CoV-2 are cough, fever and fatigue, and there are many asymptomatic infections. The most common complications in COVID-19 were acute respiratory distress syndrome (RDS), arrhythmia and shock, accounting for 25% to 35% of all complications [3,4,5]. Older people with underlying diseases such as hypertension, liver, kidney, and cardiovascular diseases have higher mortality rates [5,6]. There is increasing evidence that mortality rate in patients with COVID-19 is associated with cardiovascular comorbidities, and some possible mechanisms have been proposed.

The increased risk of arrhythmia is an important aspect of heart injury. At present, many studies have reported the correlation between COVID-19 and cardiovascular system. This review will summarize the incidence, potential mechanism, and related management measures of arrhythmia in patients with COVID-19 and provide a reference for risk relief and management of arrhythmia under the epidemic situation of COVID-19.

## 2. Incidence and Clinical Manifestations of COVID-19 Complicated with Arrhythmia

### 2.1. Incidence of Disease

In the past, severe acute respiratory syndrome virus (SARS-CoV) and Middle East respiratory syndrome coronavirus (MERS) were also coronaviruses. In the coronavirus pandemics caused by SARS-CoV and MERS, myocardial infarction, acute myocarditis, heart failure and other cardiac manifestations were reported [5]. Therefore, researchers speculated that these complications may also be associated with COVID-19 [5]. In fact, several retrospective studies have confirmed a correlation between cardiovascular risk factors and the prognosis and mortality of COVID-19. One of the most important risk factors for cardiovascular diseases in patients with COVID-19 is cardiovascular disease that exists before infection [7].

In the largest available worldwide survey [8], which involves 4526 hospitalized COVID-19 patients throughout the world, 18.27% of patients developed arrhythmias because of COVID-19. In these patients, 81.8% developed atrial arrhythmias, 20.7% developed ventricular arrhythmias, and the vast majority were tachyarrhythmias and only 22.6% were bradyarrhythmias. The results were not very different from other small and medium-sized studies in the past, where the incidence of arrhythmia in COVID-19 patients ranges from 10% to 20%. In a retrospective study in Hubei, China, nearly 7.3% of people reported palpitation as the first symptom [9]. The study of Zhao et al. [10] showed that myocardial injury is the most common cardiovascular complications in patients with COVID-19 (21.2%), followed by arrhythmia (15.3%), heart failure (14.4%) and acute coronary syndrome (1.0%). In the death cases, the combined incidence of arrhythmia, heart failure and myocardial injury was 47.8%, 40.3% and 61.7%, respectively. Sahranavard et al.’s analysis of 22 studies in 4157 patients found that the incidence of arrhythmias was 10.11% [11]. In a study involving 138 patients with COVID-19, arrhythmias accounted for 19.6% of all complications, and the incidence of ventricular tachycardia or ventricular fibrillation (malignant ventricular arrhythmias) was 5.9% [12]. At the same time, several studies have found that the incidence of arrhythmias in critically ill patients is higher than that in noncritical patients [13]. The incidence of arrhythmia in ICU patients was 37%, of which 68% were new [14], similar to the results of Bhatia et al. (43%) [15].

### 2.2. Clinical Manifestation

There are many manifestations of arrhythmia in patients with COVID-19. Most studies did not record the specific causes and types of such arrhythmias, but relevant records and data are constantly emerging. For example, in a retrospective cohort study involving 393 cases in the United States, 7.1% of patients had atrial arrhythmia [16], while in another study involving 115 cases, 16.5% of patients had atrial arrhythmia [17]. A series of cases of arrhythmia caused by SARS-CoV-2 infection showed high-grade atrioventricular block, new atrial fibrillation, long-short sequence polymorphic ventricular tachycardia caused by ventricular premature beats, and cardiac arrest with pulseless electrical activity [18]. A retrospective study of 187 patients [19] found that mortality in COVID-19 patients was associated with cerebrovascular diseases and myocardial injury, and 5.9% of these patients had ventricular tachycardia or ventricular fibrillation.

Studies have shown that the most common tachyarrhythmia in COVID-19 patients is atrial fibrillation [20], and the most common bradyarrhythmias are severe sinus bradycardia and complete heart block [20]. Tachyarrhythmia such as atrial flutter, ventricular tachycardia, atrial fibrillation, and supraventricular tachycardia has been reported in some literature. Multiple meta-analyses and studies have found that the incidence of atrial fibrillation in COVID-19 patients was around 8–13% [21,22,23], particularly common in elderly and seriously ill patients [22]. This is similar to the incidence of atrial fibrillation in influenza patients (12%) [23], so it can be speculated that atrial fibrillation is not specific to COVID-19 patients and that it is probably a common complication of the systemic inflammatory response in viral infection.

In addition, the probability of supraventricular tachyarrhythmia in severe COVID-19 patients was high, and atrial fibrillation may be a sign of critical illness, which was the most common arrhythmia in critical nursing environments [18,20].

A meta-analysis [24] that included 28 studies showed that the most common arrhythmias were supraventricular arrhythmias (6.2%) and ventricular arrhythmias (2.5%). Their study also noted that COVID-19 patients were prone to exhibit ECG features such as QTc prolongation (12.3%) and ST-segment deviation (8.7%), but the prognostic significance of these features is unclear [24]. This study result is like a retrospective study that counted data from COVID-19 patients with concomitant arrhythmias worldwide, which noted that 81.8% of those who developed arrhythmias has atrial arrhythmias, including atrial fibrillation, atrial flutter and supraventricular tachycardia [8]. 

Meanwhile, although tachyarrhythmias are the most common, 22.6% of patients may still develop bradyarrhythmias, such as bradycardia and atrioventricular block [8]. In the study by Lao et al. [25], the incidence of new-onset atrioventricular block in COVID-19 patients was 5.5%, most of which were benign. However, the study by Qingxing Chen et al. [26] concluded that ventricular tachycardia and atrioventricular block are uncommon and are most often seen at the end stage of disease in critically ill patients, and they suggested that these types of arrhythmias may serve as a sign of disease progression. For instance, a male with COVID-19 symptoms showed atrioventricular block at 2:1 and was subsequently confirmed to be infected [27]. Bradycardia has also been reported in some literature. One case of transient atrioventricular block is considered to be caused by subclinical myocarditis [28], while another case with transient complete cardiac block also developed transient S1Q3T3, suggesting that his arrhythmia may have been caused by transient pulmonary hypertension induced by acute respiratory distress syndrome [29].

## 3. Potential Mechanism of COVID-19 Complicated with Arrhythmia

Multiple trigger factors and potential mechanisms raise the risk of arrhythmias in patients with COVID-19. For COVID-19 patients with previous cardiovascular diseases, viral infection increases metabolic needs and makes chronic cardiovascular diseases unstable [30], thereby directly causing myocardial damage or inflammatory response and higher risk of arrhythmias or other heart injuries [31,32]. Current studies have suggested that patients with COVID-19 who do not have a history of cardiovascular disease also have arrhythmias and other heart damage [33,34].

**Figure 1:**
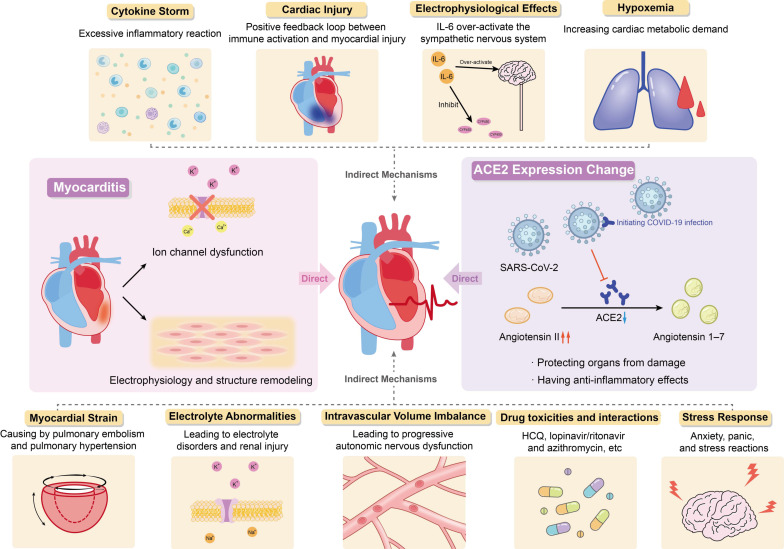
Potential Mechanism of COVID-19 Complicated with Arrhythmia.

In summary, current studies have shown that the occurrence of arrhythmias in patients with COVID-19 may be attributed to changes in angiotensin converting enzyme 2 expression, myocarditis, cytokine storm, hypoxemia, myocardial strain, electrolyte abnormalities, intravascular volume imbalance, drug interaction and stress response caused by viral infection (Figure 1).

### 3.1. Direct Mechanism

#### 3.1.1. Changes in Angiotensin Converting Enzyme 2 Expression Induced by Viral Infection

The exact pathophysiological mechanism of SARS-CoV-2-induced arrhythmias is not completely clear, but the existing data show that SARS-CoV-2 may be able to invade the heart and directly induce cardiac injury and arrhythmias. This effect is mainly achieved through the interaction of angiotensin 2 (ACE2), ACE2 inhibitor/receptor blocker and COVID-19.

Angiotensin I is converted to angiotensin II through angiotensin enzyme (ACE), which can be inhibited by ACE inhibitors. Angiotensin I and angiotensin II can be converted into angiotensin 1–9 and angiotensin 1–7 by ACE2, respectively. They can antagonize the activation of renin-angiotensin-aldosterone system (RAAS), protect organs from injury, and have anti-inflammatory effects, especially in people with history of hypertension, diabetes, or cardiovascular disease. Angiotensin II plays an inflammatory role through the angiotensin type 1 (AT1) receptor, and the combination of the two can promote the degradation of ACE2. The AT1 receptor blockers (ARBs) can inhibit this binding to prevent ACE2 degradation. Long-term use of ARBs can convert angiotensin II to angiotensin 1–7, increase the expression of ACE2 and promote anti-inflammatory effects.

The angiotensin enzyme2 is highly expressed in lung (mainly type II alveolar cells) and heart tissues and is the receptor of SARS-CoV-2 invading host cells [12,35]. The SARS-CoV-2 replicates and proliferates in cells and downregulates the expression of ACE2, which has a direct effect on the myocardium. At the same time, the virus in cells also inhibits the formation of stress granules through its accessory proteins, promotes its replication and causes cell injury [36]. This may be one of the reasons for the adverse outcome of COVID-19 patients.

Previous cardiovascular diseases affect ACE2 levels through RAAS-related pathophysiology and RAAS inhibitors [37], which will not only increase the degree of COVID-19 entering the lung and heart but also have a direct impact on cardiovascular disease, leading to heart injury. At the same time, it is also related to the poor prognosis and higher risk of death in patients with COVID-19 [3,34]. High angiotensin II levels in the circulatory system can lead to inflammation, vasoconstriction, myocardial injury, and thrombosis [33,38,39].

Therefore, ACE2 has dual effects. On the one hand, due to the high expression of ACE2 in alveolar cells and can protect lung function, long-term use of RAAS inhibitors in patients with cardiovascular disease may lead to ACE2 activity increased, which is beneficial to patients with COVID-19. On the other hand, ACE2 can initiate SARS-CoV-2 infection and then lead to downregulation of ACE2 and the toxicity of excessive accumulation of angiotensin II, gradually resulting in ARDS and fulminant myocarditis. These two effects are antagonistic to each other. Inhibition of RAAS leads to the upregulation of ACE2, which makes patients susceptible to COVID-19 but at the same time weakens the downregulation of ACE2 caused by viral infection and reduces toxicity, thus reducing the cardiovascular complications of COVID-19 patients [37,40]. At present, the potential benefits and disadvantages of ACE inhibitors/ARBs are still controversial.

#### 3.1.2. Myocarditis

Some COVID-19 patients die of fulminant myocarditis or viral activated cytokine storms, indicating that COVID-19 may cause a strong inflammatory response and increase myocarditis-related cardiac injury [41]. Viral infection is one of the leading causes of myocarditis and is also recognized in COVID-19. To date, there have been several relevant literature reports on SARS-CoV-2-induced myocarditis [42,43]. It is important to note that direct detection of the virus in the heart is only rarely reported, localization of SARS-CoV-2 is mostly confined to interstitial cells or macrophages, rather than the cardiomyocytes, meanwhile, data from Lindner et al. suggest that the presence of viruses in cardiac tissue does not necessarily cause myocarditis associated with inflammatory cell infiltration [44,45]. Recently published literature has found that the prevalence of myocarditis in autopsy cases is less than 2% [46], and the US Centers for Disease Control and Prevention has reported that although the risk of myocarditis is nearly 16 times higher in patients with COVID-19 than in those without COVID-19 and this risk is age-related, the prevalence of myocarditis in patients with COVID-19 is less than 0.2% of all cases, which is apparently very low [47]. The rate of cardiac injury in COVID-19 patients varied considerably between studies (5–40%), but overall there was a high prevalence of cardiac injury in COVID-19 patients, which is unlikely to be due to myocarditis that has a low prevalence [48]. Myocarditis associated with viral invasion, ion channel dysfunction, electrophysiology and structural remodeling caused by host and viral factors may lead to fatal arrhythmias. The mechanism is to induce abnormal calcium treatment and downregulation of potassium channel expression, resulting in prolonged repolarization time and abnormal conduction [49].

In addition, it has been reported that patients with higher levels of cardiac troponin (Tn) have a higher incidence and mortality of malignant tachyarrhythmia [19]. A study of 187 patients with COVID-19 found that 27.8% of the patients had myocardial damage, among which patients with potential cardiovascular disease with elevated troponin T (TnT) had the highest mortality, followed by patients without history of cardiovascular disease but with elevated TnT, and patients with normal TnT had the lowest mortality [19]. Serum cardiac troponin I (cTnI) levels are increased in hospitalized patients and can be used as an indicator of severe SARS-CoV-2 infection and a marker of complications caused by SARS-CoV-2 infection [50]. When the level of serum hypersensitive cardiac troponin I (hs-TnI) exceeds the upper limit of the reference range, cardiac injury is considered to occur (>28 pg/mL) [51]. A study of 671 cases showed that the level of high sensitivity plasma troponin I (hs-TnI) in 15.8% of COVID-19 patients was higher than that in the normal range, while the average level of hs-TnI in patients who died was significantly higher than that in survivors [52]. Patients with malignant tachyarrhythmia associated with elevated troponin levels should be highly suspected of having potential myocarditis [53]. The increase in troponin is often accompanied by an increase in inflammatory markers, which also suggests the role of cytokine storms in arrhythmias [54].

### 3.2. Indirect Mechanism

#### 3.2.1. Cytokine Storm

Some researchers have suggested that inflammatory cytokines may mediate the development of arrhythmias in COVID-19 patients, and that arrhythmias are not a direct result of SARS-CoV-2 infection, but rather a generalized response to the systemic inflammatory response to severe viral disease [23,55,56]. Postmortem examination showed that the myocardium of patients with COVID-19 was infiltrated by interstitial monocytes, especially in the case of fulminant myocarditis [57,58]. It can be speculated that arrhythmia is related to cytokine storm, and its essence is excessive inflammatory reaction. However, autopsy cases have also reported that the presence of the virus was not associated with increased infiltration of monocytes into the myocardium [44].

The virus entered type II alveolar cells and replicated at the early stage of SARS-CoV-2 infection. The body activates the immune inflammatory response under the effect of viral cytotoxicity, which leads to ARDS and hypoxia. In addition, the level of inflammatory cytokines in the plasma of patients in ICU and arrhythmia incidence were higher than that of patients not in ICU [19,50,59], inflammatory cytokines, such as IL-6 and IL-8, are associated with lung injury and poor prognosis in patients with COVID-19, and IL-6 and IL-10 are significantly elevated in patients with severe COVID-19 pneumonia, suggesting that the inflammatory response was related to the severity of infection and impacts arrhythmias occurrence [55]. If the virus is not effectively cleared at this stage, an excessive inflammatory response will occur. At this time, the cytokine storm will lead to multiple organ dysfunction and then arrhythmia [60]. The report showed that Th17 cells increased, CD8+ T lymphocyte toxicity increased and migration to cardiomyocytes caused inflammation in patients with COVID-19 [57]. It can be inferred that in the stage of SARS-CoV-2 infection, immune system dysfunction leads to the release of inflammatory cytokines and severe acute systemic inflammation and cytokine storm responses, resulting in irreversible multiple organ damage, including cardiac dysfunction.

#### 3.2.2. Cardiac Injury

It is well known that the inflammatory response helps to prevent infection, but when inflammatory cytokines are released excessively, an excessive inflammatory response will occur. Several studies have found that cardiac injury is common in patients with COVID-19 [61,62].

Heart damage may be a secondary manifestation of systemic infection. The release of inflammatory cytokines causes cytokine storm, which enhances the activation of T lymphocytes, thereby releasing more cytokines, forming a positive feedback loop between immune activation and myocardial injury [42]. This will strengthen myocardial injury and directly cause arrhythmias due to cellular lesions, as well as reduced coronary blood flow, reduced oxygen supply, unstable coronary plaques and microthrombosis [50]. This is also supported by the study by Xia et al. who found that elevated white blood cell counts, and elevated levels of inflammatory cytokines were more common in patients with cardiac injury and that inflammatory cytokine levels were positively associated with the risk of patients developing myocardial injury [62]. Meanwhile, the levels of circulating pro-inflammatory cytokines in COVID-19 patients are directly and independently correlated with troponin levels [63].

#### 3.2.3. Electrophysiological Effects

Systemic inflammation rapidly induces cytokine-mediated electrical remodeling of the ventricles and significant QTc prolongation during acute infections [64]. Previous studies have found that IL-6 can directly block hERG channels in ventricular myocytes and over-activate the sympathetic nervous system [65,66]. A large cohort study showing an independent association between SARS-CoV-2 infection and QTc prolongation, IL-6 levels and QTc maximum in hospitalized COVID-19 patient were directly correlated, suggesting that IL-6 and QTc prolongation are related, but other factors may be involved [67]. Other studies have similar findings, with elevated IL-6 predicting the risk of arrhythmias in COVID-19 patients [55,68].

Tumor necrosis factor-α (IL-1), IL-6 (IL-6) and IL-1 (IL-1) have also been shown to regulate the function of ion channels and prolong the time of ventricular action potential by interacting with potassium and calcium channels [64,69,70]. Moreover, elevated cytokines (especially IL-6) also reversibly downregulate the expression of connexin43 in the atria and atrioventricular node, rapidly inducing cardiac electrical remodeling and increasing atrial fibrosis, thereby promoting the development of atrial fibrillation and AV block [71,72].

In addition, increased IL-6 during systemic inflammation can inhibit cytochrome P450, especially CYP3A4, which may lead to the QT prolongation and increase the danger of torsional ventricular tachycardia at the tip [73,74,75]. The SARS-CoV-2 had subtype-specific effects on CYP, such as reduced activity of CYP1A2, CYP2C19 and CYP3A and increased activity of CYP2B6 and CYP2C9 [75,76].

Besides effects on CYP, other electrophysiological effects caused by cytokines also have effects on the organism. The presence of fever in COVID-19 patients may promote the development of LQTS by altering K+ dependence [77]. The inflammatory response releases large amounts of inflammatory cytokines that act on the hypothalamus, leading to activation of the sympathetic nervous system via the central pathway and consequent arrhythmias [78]. Inflammatory factors acting on the peripheral pathway lead to increased left stellate ganglion remodeling, which also leads to the same result [79]. In the male, a systemic inflammatory response is associated with reduced testosterone levels and transient inflammatory hypotestosteronemia, which is significantly associated with an increased risk of long QT syndrome and TdP [80].

#### 3.2.4. Hypoxemia

The SARS-CoV-2 infection can cause severe lung injury, resulting in acute respiratory failure and myocardial damage. According to a previous study, 32% of COVID-19 patients develop different degrees of hypoxemia [81]. Up to 76% of patients need oxygen support [59]. Hypoxemia may increase pulmonary artery pressure and right ventricular afterload, increase cardiac metabolic demand, affect myocardial oxygen supply, and generate myocardial injury [82,83]. The study by Guo T. et al. also confirmed that COVID-19 patients with myocardial injury have worse hypoxemia compared to those without myocardial injury [19].

#### 3.2.5. Myocardial Strain

Pulmonary embolism is one of the most common thrombotic complications in COVID-19 patients [84], and patients presenting with ARDS can develop secondary pulmonary hypertension. Pulmonary embolism and pulmonary hypertension may cause myocardial strain. On the one hand, a COVID-19 patient with transient pulmonary hypertension developed transient complete cardiac block [29]. On the other hand, patients with pulmonary hypertension and hypoxemia have higher risk of atrial tachyarrhythmia on account of right atrial pressure and sympathetic nerve excitement [85]. The degree of myocardial strain is associated with the development of QTc prolongation in COVID-19 patients, which can be indicated by circulating BNP levels [86].

#### 3.2.6. Electrolyte Abnormalities

Arrhythmia caused or aggravated by abnormal electrolytes in patients with COVID-19 has been reported to a certain extent [87,88]. Electrolyte disturbances are common in COVID-19 patients, particularly hypokalaemia, hyponatraemia and hypocalcaemia. In a case series containing 416 patients, 7.2% of patients were reported electrolyte disorders [89]. Altered function of the RAAS system, gastrointestinal dysfunction, inflammatory factors and secondary renal tubular dysfunction caused by viral invasion may all contribute to electrolyte imbalance [90]. A retrospective study of 85 patients showed that 27% of patients had acute renal injury [91].

#### 3.2.7. Intravascular Volume Imbalance

Intravascular volume imbalance is a common symptom in severely ill patients. COVID-19 patients may have an imbalance in intravascular volume due to sepsis or heart failure caused by ARDS, which will lead to increased sympathetic activity and arrhythmia [92]. The most common type of arrhythmia in severe patients is supraventricular arrhythmia, especially atrial fibrillation. This is not only because of the above mechanism of intravascular volume imbalance but also because of the increase in catecholamine release and progressive autonomic nervous dysfunction, anemia, pain, excitement, ventilator synchronization, hypoxia, hypercapnia, acidosis and so on, which may also lead to arrhythmia [93].

#### 3.2.8. Drug Toxicities and Interactions

The occurrence of arrhythmia in COVID-19 patients can be also the result of drugs toxicities and interactions. In antiviral therapy, doctors may use multiple drugs at the same time, and the interaction of drugs may increase the risk of arrhythmias.

More adverse reaction of drugs during the treatment of COVID-19 have been reported. The commonly used hydroxychloroquine (HCQ), lopinavir/ritonavir and azithromycin have effects on the prolongation of QT and increase the danger of Torsades de Pointes ventricular tachycardia (TDP) [81,94].

Antimalarials, lopinavir/ritonavir and azithromycin can inhibit hERG-K+ channels, leading to prolonged action potentials and QT intervals and serious arrhythmias such as ventricular tachycardia and even ventricular fibrillation, which increase the probability of TDP in patients with an arrhythmia tendency [95,96,97]. Both azithromycin and Remdesivir have been reported to have harmful effects on the cardiovascular system [98,99]. A retrospective cohort study of 40 COVID-19 patients showed that QTc prolongation occurred in six (33%) of 18 patients treated with hydroxychloroquine and azithromycin, while QTc prolongation occurred in only one (5%) of 22 patients treated with hydroxychloroquine alone [66].

Among the adverse reactions to hydroxychloroquine reported in 2020, the most common were diarrhea, prolonged QT interval and hypertransamininaemia, the severity of which was related to the dose of hydroxychloroquine used [100]. At the same time, hydroxychloroquine not only causes the fatal effect of prolonging QT but also leads to atrioventricular block [32]. Among other agents used for the treatment of COVID-19, azithromycin use in combination with other drugs was found to serve as a predictor of serious adverse events [100]. In study reported by Mercuro, the combination of hydroxychloroquine and azithromycin may lead to prolonged QTc and increased risk of TdP [101].

However, Rosenberg et al. [102] reported a multicenter retrospective study of 1438 patients with COVID-19, which showed that hydroxychloroquine was not associated with in-hospital mortality. There was no significant difference in the mortality of COVID-19 patients treated with hydroxychloroquine, azithromycin, or the combination of the two in the ICU. These studies were carried out in an ICU environment where QTc was continuously monitored, and the significance of QTc extension in outpatients or ordinary inpatients cannot be determined. However, although some studies have concluded that the combination of hydroxychloroquine and azithromycin does not contribute significantly to arrhythmias, there is evidence that COVID-19 patients with high levels of IL-6 are more likely to develop arrhythmias when this treatment is used [103]. Excessive conduction abnormalities, prolongation of QTc and APD may lead to fatal arrhythmias [103]. Overall, using hydroxychloroquine and azithromycin as the treatment of COVID-19 remains controversial [104]. Therefore, in the presence of elevated IL-6, the use of potentially fatal arrhythmic drugs must be closely monitored.

#### 3.2.9. Stress Response

In the face of sudden outbreaks and widespread outbreaks, COVID-19 patients tend to bear great psychological pressure, and they will be more likely to feel anxiety, panic, and develop stress-related reactions. Under the influence of the stress state and side effects of drugs, the body will secrete a large number of catecholamines, resulting in vasoconstriction and cardiotoxicity [105], leading to myocardial injury or malignant arrhythmias.

## 4. Management Scheme of Arrhythmia Treatment in the Presence of COVID-19

With the continuous spread of COVID-19, cardiovascular complications are becoming increasingly common. Even in patients without previous cardiovascular disease, there is a risk of cardiovascular complications, disease development and prognosis are also affected. Therefore, considering the high incidence of arrhythmia and its influence on prognosis, it is necessary to study the details and specific mechanisms of arrhythmia types in patients with COVID-19, which may be helpful for clinicians to manage the prognosis of patients. On the other hand, in addition to the basic detection, the heart condition of COVID-19 patients and the heart damage related to virus infection should be detected, and appropriate management strategies should be adopted to minimize the adverse effects of arrhythmia and SARS-CoV-2 infection as much as possible. The management of arrhythmias in patients with COVID-19 is mainly carried out by reducing virus exposure and paying attention to drug interactions.

### 4.1. Antiviral Therapy

At present, the treatment of COVID-19 has not been standardized, most of which are symptomatic. Several drugs are being used or being considered for COVID-19. The Infectious Diseases Society of America proposed seven possible therapies for COVID-19 in the guidelines [106], including hydroxychloroquine/chloroquine, hydroxychloroquine/chloroquine plus azithromycin, the combination of lopinavir/ritonavir, and the use and against the use of corticosteroids, tocilizumab, and COVID-19 convalescent plasma. Among them, hydroxychloroquine/chloroquine plus azithromycin, the combination of lopinavir/ritonavir, tocilizumab, and COVID-19 convalescent plasma are recommended only in clinical trial, and corticosteroids use depends on whether the COVID-19 patients have ARDS or not. As mentioned above, IL-6 was significantly increased in patients with COVID-19 and was associated with arrhythmia [103]. Anti-IL-6 receptor monoclonal-antibody tocilizumab can reduce the prolongation of QT by IL-6 [107]. At the same time, IL-6 can inhibit cytochrome P450, which can increase bioavailability of several QT-prolonging drugs and induce the activation of sympathetic system, resulting in long QT syndrome and various arrhythmias [75,107]. Other IL-6 therapies also have potential antiarrhythmic activities [107].

Although dexamethasone and other drugs have been shown to reduce the mortality of hospitalized COVID-19 patients [108], a larger randomized controlled trial is still needed to further elucidate the efficacy and optimal use of the relevant drugs against SARS-CoV-2. Many hospitals use hydroxychloroquine, lopinavir/ritonavir, and azithromycin, but their effectiveness and safety are still controversial [109]. It is also important to note that high doses of chloroquine may result in increased mortality and prolonged QT intervals in life-threatening situations compared to low doses of chloroquine, suggesting that appropriate dose studies are needed before applying drugs to the treatment of COVID-19 [110]. A meta-analysis of 27 randomized controlled trials showed that compared with routine nursing, IL-6 antagonist significantly reduced the short-term mortality of COVID-19 patients, which was more obvious in patients receiving corticosteroids [111]. It is well known that corticosteroids have a good anti-inflammatory effect. Studies have shown that corticosteroids therapy can also improve the mechanical ventilation of severe COVID-19 patients and has lower mortality than patients with routine nursing [112]. In short, there are still many clinical studies related to antiviral therapy. The following table summarizes some related clinical research progress (Table 1).

### 4.2. The Method of Dealing with Tachyarrhythmia

For COVID-19 patients with arrhythmia symptoms, echocardiography should be used to assess myocardial function and detect abnormal local wall contraction, acute valvular disease, and noninvasive hemodynamics [136].

For COVID-19 patients diagnosed with arrhythmia, there is not enough clinical research to guide the treatment. Therefore, it should be managed according to standard guidelines, which are generally similar to the treatment of arrhythmias caused by infection or transient metabolic disorder. The focus is on the temporary rhythm, drug interaction, reducing exposure and strengthening personal protection. Considering the possibility of hemodynamic instability and thrombus embolism, antiarrhythmic drugs, electrocardioversion and anticoagulation are involved in the treatment. Prolonged prothrombin time is also one of the most common features of patients with COVID-19, therefore, more caution needed when using anticoagulants [137]. Corticosteroids also need to be considered to reduce possible inflammatory reactions in patients with COVID-19 [138]. At the same time, it is necessary to conduct risk-benefit assessments, postpone nonemergency procedures, and conduct electrophysiological studies on refractory and life-threatening arrhythmia patients [88].

The interaction of some drugs can easily lead to bradycardia in COVID-19 patients, such as bradycardia caused by sinus bradycardia or atrioventricular block. Isoproterenol and atropine may be considered first in patients with persistent bradycardia [139]. Universal pacemaker therapy should be carried out for all clinically meaningful chronic tachyarrhythmia, regardless of how the virus infects [140]. At the same time, considering the transient arrhythmias caused by viral infection, as well as the possible bacterial overlapping infection, the temporary pacemaker should be implanted preferentially. After the recovery of COVID-19, the cardiac function of the patients should be reevaluated, and permanent pacemakers should be considered according to the results of the evaluation [141].

For patients with atrial tachyarrhythmias such as supraventricular tachycardia, atrial fibrillation or flutter, the identification of its secondary causes is very important. The hypoxia, electrolyte abnormalities, drug interactions and so on mentioned above may lead to this kind of arrhythmia. Therefore, it is necessary to find the trigger and correct it in time, supplemented by the commonly used treatment methods of tachyarrhythmia. Intravenous adenosine may be considered in patients with supraventricular tachycardia, and if it is difficult to correct, electrocardioversion can be considered. For patients treated with β-receptor blockers (BBS) or calcium channel blockers (CCBS), on the one hand, it is necessary to consider whether there is contraindication; on the other hand, they need to evaluate the interaction between these drugs and antiviral drugs to avoid bradycardia or prolonged QT [139,141]. For patients with atrial fibrillation, it is necessary to determine whether it is recurrent atrial fibrillation or new-onset atrial fibrillation. If it is a patient with recurrent atrial fibrillation, it is necessary to consider that antiarrhythmic drugs such assotalol may interact with antiviral drugs and give BBS or CCBS such heart rate control drugs for treatment. Similarly, heart rate control strategies can be used in patients with newly occurring atrial fibrillation or flutter with stable rhythm. However, if the cause of arrhythmia is not corrected in time, the risk of recurrence is high, so timely and targeted treatment is needed to minimize the risk [139,141]. In addition, it should be emphasized that there are many data indicating that SARS-CoV-2 infection may increase the risk of thrombosis, so for such patients, it is necessary to assess the risk of thromboembolism during and after infection recovery and to improve anticoagulant strategies [142].

Similar to patients with atrial tachyarrhythmia, the treatment of patients with ventricular arrhythmia is also focused on identifying and correcting possible secondary causes such as hypoxemia, electrolyte abnormalities and drug interactions.

For COVID-19 patients with channel lesions or hereditary arrhythmia syndrome, particular attention should be given to their cardiovascular conditions. Patients with COVID-19 have a high risk of prolonged QT. For patients with long QT syndrome (LQTS), QT interval and rhythm need to be monitored. As the increased release of catecholamine may lead to arrhythmias, the injection of catecholamine should be more cautious for critically ill patients with epinephrine-related ventricular arrhythmia [93].

### 4.3. Optimal Management

Considering the high hospitalization and mortality rate of patients in the ICU with COVID-19 complicated with cardiovascular disease, special consideration should be provided for arrhythmia in these patients with preexisting cardiovascular disease while receiving COVID-19 treatment, and the prolongation of the QTc interval should be strictly detected [143]. In addition, although patients with COVID-19 combined with atrial fibrillation have a higher risk of adverse outcomes than patients without atrial fibrillation, whereas patients with atrial fibrillation on long-term anticoagulants prior to SARS-CoV-2 infection are at lower risk of death, so knowledge of the patient’s previous cardiovascular disease treatment is also warranted [144].

For patients with general COVID-19, echocardiography and troponin should be avoided as routine examinations of COVID-19 patients, but other cardiac biomarkers, such as NT-proBNP and electrocardiogram, should be closely detected to intervene in time. Once relevant clinical features appear, echocardiography and troponin examination are then used to aid in the diagnosis, management, and prognosis of cardiovascular diseases [145].

For medical workers who are often required to be in close contact with COVID-19 patients, they should take strict preventive measures and use personal protective equipment effectively to prevent themselves from being infected. Hospitals need to optimize the triage process to reduce the flow of COVID-19 patients. It is also essential to minimize the risk of SARS-CoV-2 infection through stratified intervention and telemedicine.

## 5. The Prognostic Impact of COVID-19 on Arrhythmia

Although current studies have shown that COVID-19 may cause arrhythmias due to changes in angiotensin converting enzyme 2 expression, myocarditis, cytokine storms, hypoxemia, myocardial strain, electrolyte abnormalities, intravascular volume imbalance, drug interaction and stress response caused by viral infection, it is impossible to follow up with patients with arrhythmia due to the length of the pandemic being less than three years.

The ECG is strongly correlated with the severity of SARS-CoV-2 infection. For short-term prognosis, one study found that cardiac injury, as assessed by troponin, was associated with short-term mortality [41,146]. In contrast, a standard ECG obtained on admission to hospital can predict inpatients with a poor short-term prognosis by showing acute changes and chronic abnormalities [147,148]. A continuous ECG during hospitalization can provide better stratification of patient prognosis [149].

There is no clear evidence of long-term prognostic implications. However, the inflammation, immune system disorder and myocardial injury caused by virus attack may cause irreversible damage to the cardiovascular system [41,62,78]. In the future, these injuries will increase the probability of cardiovascular disease in patients and may also aggravate the original cardiovascular disease condition of patients [42]. Therefore, although there are no clear data, it is not difficult to predict that the long-term prognosis of patients will be adversely affected, which also suggests that while paying attention to the potential mechanism of COVID-19 complicated with arrhythmia, we should also explore and study the possible prognostic outcomes and further do a good job of prevention and management during the epidemic situation.

## 6. Discussion and Prospect

Today, the global epidemic situation is still serious. With the development of research, cardiovascular injury in patients with COVID-19 is the second major symptom in addition to respiratory symptoms. The increased risk of arrhythmia is an important aspect of heart injury. According to previous research, people know more about the direct and indirect mechanisms by which arrhythmias occur in patients with COVID-19. This review overviewed the clinical manifestations of COVID-19 complicated with arrhythmia and summarized the potential mechanisms, and suggested the management scheme of arrhythmia treatment against a background of COVID-19.

The SARS-CoV-2 has severe effects on the cardiovascular system. Clinicians need to focus upon the cardiovascular system of COVID-19 patients and treat them in a timely manner to avoid the adverse consequences caused by cardiovascular complications. Currently, the understanding of the pathophysiological mechanism of SARS-CoV-2 affecting cardiac function is still very limited, and further exploration is needed. The combination of drugs in clinical treatment also needs to be considered. On the one hand, there is a lack of research reports on the specific types of arrhythmias; on the other hand, almost all the existing studies are retrospective, and there is a lack of randomized controlled studies to guide the clinical setting. In addition, future studies also need to pay attention to how to improve and improve the outcome of COVID-19 complicated with arrhythmia and the quality of life of patients.

In summary, with the continuous pandemic of SARS-CoV-2 infection, to better prevent and treat arrhythmia in patients with COVID-19, it is necessary to describe the mechanism of arrhythmia in patients with COVID-19 in a more specific way, to improve the understanding of potential drugs and to verify and optimize the relevant management strategies to provide new ideas for clinical practice.

## Figures and Tables

**Table 1:** Summary of Some Related Clinical Research Progress.

First Author	Year	Country	Study Type	Study Duration	Participants	Treatment	Result
Antinori et al. [113]	2020	Italy	Prospective Study	23 February–20 March 2020	35	Remdesivir	Ramidivir is effective for hospitalized patients with COVID-19 with few adverse reactions.
Olender et al. [114]	2021	Multinational	Randomize Phase III Clinical Trial and Retrospective Cohort Study	9 March–10 April 2020	1130	Remdesivir	Remdesivir is associated with greater recovery in patients with severe COVID-19.
Spinner et al. [115]	2020	the US, Europe, and Asia	Controlled Trial	15 March–18 April 2020	596	Remdesivir	No significant difference in the additional therapeutic effect of patients with moderate COVID-19 treated with remdesivir.
Wang et al. [116]	2020	China	RCT	6 February–12 March 2020	237	Remdesivir	Remdesivir was not associated with statistically significant clinical benefits.
Cao et al. [117]	2020	China	RCT	18 January–3 February 2020	199	Lopinavir-Ritonavir	No additional benefit was observed in patients treated with lopinavir-ritonavir beyond the usual care.
Reis et al. [118]	2021	Brazil	RCT	2 June–30 September 2020	685	Hydroxychloroquine or Lopinavir-Ritonavir	No important clinical benefits of hydroxychloroquine or lopinavir-ritonavir were discovered.
Mitjà et al. [119]	2021	Spain	RCT	17 March–26 May 2020	293	Hydroxychloroquine	No additional benefit was observed in patients treated with HCQ beyond the usual care.
Skipper et al. [120]	2020	the US and Canada	RCT	22 March–20 May 2020	491	Hydroxychloroquine	Hydroxychloroquine did not substantially reduce symptom severity in outpatients with early, mild COVID-19.
Aman et al. [121]	2021	Netherlands	RCT	31 March–4 January 2021	400	Imatinib	Imatinib may benefit in COVID-19 patients, safety evaluation showed no adverse events related to imatinib.
Caplan et al. [122]	2021	France	Single center Retrospective Study	27 February–14 April 2020	169	Almitrine Infusion	Almitrine infusion improved oxygenation in COVID-19 patients without adverse effects.
Ochoa et al. [123]	2021	Mexico	Prospective Trial	5 June–5 August 2020	243	Sulodexide	Sulodexide was effective in reducing hospitalization time and supplemental oxygen treatment, and had no obvious side effects.
Moragón et al. [124]	2021	Spain	RCT	19 October–19 January 2021	20	Metoprolol	Intravenous metoprolol reduced lung inflammation, and improved oxygenation.
Oldenburg et al. [125]	2021	the US	RCT	May–March 2021	263	Azithromycin	The results showed that single dose azithromycin might be ineffective.
Lescure et al. [126]	2021	Multinational	RCT	28 March–3 July 2020	431	Sarilumab	The results suggest that a short course of MP in COVID-19 patients did not reduce mortality.
Ramakrishnan et al. [127]	2021	the UK	RCT	16 July–9 December 2020	167	Inhaled Budesonide	Inhaled budesonide reduced the possibility of emergency medical care and the recovery time of early COVID-19.
Feld et al. [128]	2021	Canada	RCT	18 May–4 September 2020	60	Peginterferon Lambda	Peginterferon lambda may prevent deterioration and shorten duration of viral shedding in COVID-19 patients.
Jeronimo et al. [129]	2021	Brazil	RCT	18 April–16 June 2020	647	Methylprednisolone (MP)	The results suggest that a short course of MP in COVID-19 patients did not reduce mortality.
Hung et al. [130]	2020	China	Multicenter Prospective Study	10 February–20 March 2020	127	Triple Combination of Interferon Beta-1b, Lopinavir-Ritonavir, and Ribavirin	Early triple antiviral therapy was safe and superior to lopinavir–ritonavir alone in COVID-19 patients.
Meng et al. [131]	2020	China	Non-randomized Phase I Clinical Trial	27 January–30 March 2020	18	Human Umbilical Cord-derived Mesenchymal Stem Cell Therapy (UC-MSCs)	The results suggest that intravenous UC-MSCs infusion in COVID-19 patients is safe and well tolerated.
Cao et al. [132]	2021	China	Multicenter Retrospective Study	7 February–30 March 2020	26	High-Dose Intravenous Immunoglobulin (IVIg)	High-dose IVIg treatment reduced 28-day mortality.
Balcells et al. [133]	2021	Chile	RCT	10 May–18 July 2020	57	Convalescent Plasma (CP)	No evidence showed the addition of CP in the early stages of COVID-19 differs from only in patients with deterioration.
Ameri et al. [134]	2020	Iran	Self Control Test	21 May–24 June 2020	5	Low-dose whole-lung radiation therapy (LD-RT)	LD-RT may be effective for severe COVID-19 patients.
Weinreich et al. [135]	2021	the US	RCT	16 June–13 August 2020	275	REGN-COV2	The REGN-COV2 antibody cocktail showed greater effect in patients who had not initiated immune response or had high baseline viral load.
